# Supplementary material for: Intradermal Vaccination against Influenza with a STING-Targeted Nanoparticle Combination Adjuvant Induces Superior Cross-Protective Humoral Immunity in Swine Compared with Intranasal and Intramuscular Immunization
Source: Vaccines (Basel). 2023 Nov 7;11(11):1699. doi: 10.3390/vaccines11111699 (PMC10675188; doi:10.3390/vaccines11111699)
Supplement: Supplementary file 1 [file vaccines-11-01699-s001.zip › vaccines-2683128-supplementary.pdf]

Supplemental Information

Intradermal vaccination against influenza with a STING-targeted nanoparticle combination adjuvant induces superior cross-protective humoral immunity in swine compared with intranasal and intramuscular immunization

Juan F Hernandez-Franco[1], G. Yadagiri[2], V. Patil[2], D. Bugybayeva[2], S. Dolatyabi[2], E. Dumkliang[3], Mithilesh Singh[2], R. Suresh[2], F. Akter[2], J. Schrock[2], G. J. Renukaradhya[2]*, and Harm HogenEsch[1,4]*

[1] Department of Comparative Pathobiology, College of Veterinary Medicine, Purdue University; [2] Center for Food Animal Health, Department of Animal Sciences, The Ohio State University, Wooster, OH 44691; [3] Drug Delivery System Excellence Center (DDSEC), Department of Pharmaceutical Technology, Faculty of Pharmaceutical Sciences, Prince of Songkla University, Songkhla, 90110, Thailand; [4] Purdue Institute of Inflammation, Immunology and Infectious Disease, Purdue University, West Lafayette, IN 47907.

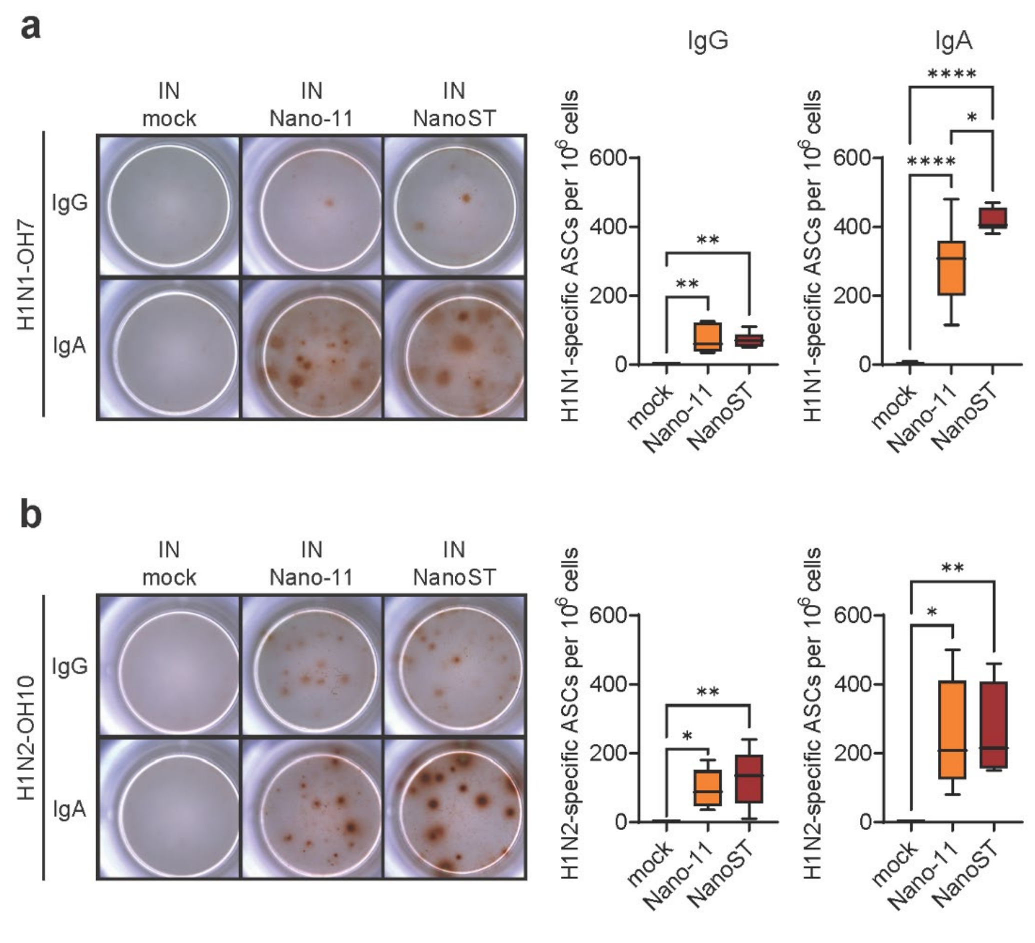

Supplemental Figure S1. Intranasal vaccination with split H1N2/NanoST stimulates the generation of influenza-specific cross-reactive antibody secreting cells (ASCs) within the bone marrow. Pigs were immunized intranasally with split H1N2 swIAV with Nano-11 or NanoST as described previously [1]. The pigs were challenged with heterologous H1N1 virus. Bone marrow cells were collected with an aspiration needle 6 days after challenge. ELISpot was utilized to quantitate the (**a**) H1N1-OH7 and (**b**) H1N2-OH10-specific IgG and IgA ASCs. Results are reported as influenza-specific ASCs/per 10^6 bone marrow-derived cells. Data represents mean value of 5 to 6 pigs ± SEM. *p <0.05, **p <0.01, ****p<0.0001 by one-way ANOVA with Tukey's multiple comparison test.

1. Patil, V.; Hernandez-Franco, J.F.; Yadagiri, G.; Bugybayeva, D.; Dolatyabi, S.; Feliciano-Ruiz, N.; Schrock, J.; Hanson, J.; Ngunjiri, J.; HogenEsch, H., et al. A split influenza vaccine formulated with a combination adjuvant composed of alpha-D-glucan nanoparticles and a STING agonist elicits cross-protective immunity in pigs. *J Nanobiotechnology* **2022**, *20*, 477, doi:10.1186/s12951-022-01677-2.
